# PAHs Target Hematopoietic Linages in Bone Marrow through Cyp1b1 Primarily in Mesenchymal Stromal Cells but Not AhR: A Reconstituted* In Vitro* Model

**DOI:** 10.1155/2016/1753491

**Published:** 2016-11-07

**Authors:** Catherine M. Rondelli, Michele Campaigne Larsen, Alhaji N'jai, Charles J. Czuprynski, Colin R. Jefcoate

**Affiliations:** ^1^Molecular and Environmental Toxicology Program, University of Wisconsin, Madison, WI, USA; ^2^Department of Cell and Regenerative Biology, University of Wisconsin Medical School, Madison, WI 53705, USA; ^3^Department of Pathobiological Sciences, University of Wisconsin, Madison, WI 53706, USA

## Abstract

7,12-Dimethylbenz(a)anthracene (DMBA) rapidly suppresses hematopoietic progenitors, measured as colony forming units (CFU), in mouse bone marrow (BM) leading to mature cell losses as replenishment fails. These losses are mediated by Cyp1b1, independent of the AhR, despite induction of Cyp1b1. BM mesenchymal progenitor cells (MPC) may mediate these responses since basal Cyp1b1 is minimally induced. PreB colony forming unit activity (PreB CFU) is lost within 24 hours in isolated BM cells (BMC) unless cocultured with cells derived from primary MPC (BMS2 line). The mouse embryonic OP9 line, which provides more efficient coculture support, shares similar induction-resistant Cyp1b1 characteristics. This OP9 support is suppressed by DMBA, which is then prevented by Cyp1b1 inhibitors. OP9-enriched medium partially sustains CFU activities but loses DMBA-mediated suppression, consistent with mediation by OP9 Cyp1b1. PreB CFU activity in BMC from Cyp1b1-ko mice has enhanced sensitivity to DMBA. BMC gene expression profiles identified cytokines and developmental factors that are substantially changed in Cyp1b1-ko mice. DMBA had few effects in WT mice but systematically modified many clustered responses in Cyp1b1-ko mice. Typical BMC AhR-responsive genes were insensitive to Cyp1b1 deletion. TCDD replicated Cyp1b1 interventions, suggesting alternative AhR mediation. Cyp1b1 also diminishes oxidative stress, a key cause of stem cell instability.

## 1. Introduction

People are chronically exposed to polycyclic aromatic hydrocarbons (PAHs) in multiple ways ranging from cigarette smoke to diesel fumes and coal tars [1]. Many PAHs are converted by metabolism at P450 cytochromes (Cyps) to highly reactive and mutagenic dihydrodiol epoxide metabolites [2]. The highest level of such metabolism is provided in the liver by cytochrome P4501A1 (Cyp1a1), which, however, also has high levels of enzymes, notably, glutathione transferases that provide protection against this toxicity. In the bone marrow (BM), there is similarly active cytochrome P450 1b1 (Cyp1b1), in close proximity to the hematopoietic stem cell niche [3]. Numerous studies in mice have shown that repeated daily administration of PAHs causes immunosuppression. Many effects of TCDD on the immune system are produced by direct activation of the aryl hydrocarbon receptor (AhR) [4]. This intervention applies to T cells, at both the level of thymus progenitors and Treg/T17 cells. PAHs, like TCDD, activate the AhR to induce Cyp1a1 and Cyp1b1 [5, 6]. Thus, PAHs can function both through activation of AhR and through their conversion to reactive metabolites. Here, we describe new approaches to resolving the impact of PAH metabolites on hematopoietic stem cells and the lineage progenitors* in vivo* and in a cell culture model.

In order to better understand the mechanism of this immunosuppression, we have used single intraperitoneal or oral doses. This approach provides better definition of the time course and individual steps that suppress immune cells. Colony forming unit (CFU) assays show that moderate single doses of two PAHs, 7,12-dimethylbenz(a)anthracene (DMBA) and benzo(a)pyrene (BP), suppress the proliferative activity of lymphoid, myeloid, and erythroid progenitors within 6 hours. Mature BM cells are unaffected as shown by minimal gene expression responses to DMBA [5, 6]. These shared progenitor suppression responses suggest that stem cell differentiation to the respective lines is blocked by DMBA metabolites. These suppressions are removed in Cyp1b1-ko mice but are surprisingly independent of the AhR, which mediates induction of Cyp1b1 by DMBA in most cell types.

BM lymphoid and myeloid cells become depleted between 24 and 48 hours after DMBA administration. During this period, lymphocytes are similarly depleted from the thymus (T cells) and spleen (B cells). Different doses and routes of DMBA administration produce 48-hour depletions of mature lymphocytes in BM, thymus, and spleen that each correlate with 6-hour suppression of BM PreB CFU expansion activity [6]. We concluded that the initial impact of DMBA on the lymphocyte populations of each tissue was caused by suppression of the BM common lymphoid progenitors (CLP) and their progression from the stem cells. This conclusion was supported by flow analyses of the CLP and other progenitor populations. This results in a failure to replace cells that are exported from these three sources, particularly to sites of injury, such as the lung.

BM mesenchymal progenitors, however, are exceptional for robust basal Cyp1b1 expression that is relatively insensitive to AhR induction. We hypothesize, therefore, that the suppression of BM progenitor activity is mediated via mesenchymal progenitors that are in close proximity to the stem cell niche. BP is distinguished from DMBA by recovery of hematopoietic activities over 24 hours, through an AhR-dependent process. This recovery is associated with large AhR-mediated increases in multiple cytokines that we attribute to hepatic metabolism of the BP following the extensive induction of Cyp1a1. This metabolism forms BP quinones, which can activate the NF-*κ*B pathway, a well-characterized route to cytokine stimulation. Among the stimulated cytokines, Il6 is known to protect hematopoietic progenitors. Cyp1 family members also intervene in the generation of inflammatory regulatory molecules (protectins, resolvins) [7].

We originally identified Cyp1b1 (then P450EF) as the AhR-induced cytochrome P450 in multipotential mouse embryo fibroblasts (MEFs) [8]. Subsequent gene cloning, first from human keratinocytes [9], and parallel efforts in MEFs [10] and rat adrenals [11] established that the same Cyp1b1 gene is involved and suggested a developmental function. The linkage of mutations to glaucoma [12] established a human physiological role. This has been further emphasized by links to cellular morphology and vascular development in endothelia and pericytes [13], to murine female hypertension [14], and to obesity and NASH in male and female mice [15]. Cyp1b1 has also been shown to be highly expressed in many tumors [16]. This role was further emphasized by the endogenous activation of Wnt-induced intestinal polyps in Cyp1b1-ko mice [17].

In order to dissect the role of Cyp1b1 metabolism of PAHs in BM hematopoiesis, we have established an* in vitro* coculture model in which primary mouse BM cells, isolated by collagenase treatment, are cocultured with mesenchymal progenitor cell (MPC) lines. The OP9 cell line, derived from the aorta-gonadal mesonephros (AGM) region of the embryo [18], is routinely used as a stromal support for stem cell cultures. Here, we demonstrate that OP9 cells effectively sustain PreB progenitor proliferative activity for 24 hours, as measured in the PreB colony forming unit assay (PreB CFU). We use this coculture model to show that DMBA and BP effectively suppress this activity. Other MPC lines function in the same way but are less efficient in sustaining CFU activity. In this work, we show that Cyp1b1 activity in the OP9 cells, rather than in the BMC, mediates the PAH suppression of CFU activity. We show that OP9-enriched media support the CFU activity but also that this coculture is no longer sensitive to PAH suppression. The use of Cyp1b1-ko BMC confirms this model and also shows that Cyp1b1 contributes to* in vitro *stability of the HSC activity and, surprisingly, enhances the sensitivity of PreB progenitors to DMBA. To address this anomaly, we examined the gene expression profiles of BM cells from WT and Cyp1b1-ko BMC, as well as the* in vivo* responses to DMBA. A set of Cyp1b1-responsive cytokine and developmental markers is identified.

## 2. Materials and Methods

### 2.1. Animals

C57BL/6J (wild type, WT) mice and AhR^d^ mice were purchased from the Jackson Laboratories (Bar Harbor, ME). Cyp1b1-null (Cyp1b1-ko) mice were bred in our animal care facility at the Biotron animal care facility [19]. All mice were housed in the AAALAC certified University of Wisconsin Madison School of Veterinary Medicine and Biotron Animal Care Units and used in accordance with the NIH Guide for the Care and Use of Laboratory Animals.

### 2.2. PAH Treatment* In Vivo*

Animals were treated with PAH, TCDD, or vehicle control (olive oil) via a single IP injection (DMBA, 50 mg/kg; BP, 50 mg/kg; TCDD, 0.03 mg/kg (Accustandard; New Haven, CT)) prior to isolation of the BMC for analysis [1, 2]. All TCDD exposures were completed in 12 hours, while PAHs were tested for up to 48 hours.

### 2.3. Bone Marrow Isolation

Mice were euthanized under CO_2_. Femurs were immediately extracted and crushed in extraction media (RMPI + 2% FBS). The bone marrow was exposed and cells were released from the matrix using collagenase type 1 (1600 U) (Worthington, Lakewood, NJ) digestion for 15 min at 37°C, with shaking at 110 rpm [6]. BMC were washed and collected in extraction media using centrifugation.

### 2.4. Cells/Cell Culture

BMS2 cells were a gift from Dr. Paul Kincade [20], while C3H10T1/2 [21] and OP9 [18] cells were purchased from ATCC (Manassas, VA). Cells were cultured under standard conditions (37°C at 5% CO_2_ in saturated atmospheric humidity), in FBS (Atlanta Biologicals, Flowery Branch, GA) supplemented media (RPMI 1640, BMS2; DMEM, C3H10T1/2; *α*MEM, OP9) (Fisher Scientific, Waltham, MA) as per recommendations, with passages at 80% confluence. Feeder stromal cultures were grown to approximately 100% confluence and treated with 10 *μ*g/mL mitomycin C (Sigma, St. Louis, MO) for 24 hours to inhibit further cellular division. Mitomycin C (2 mg/5 mL) stocks were maintained in PBS and kept frozen in liquid nitrogen. Treated feeder layers were maintained in culture for up to 6 weeks. Cocultures were comprised of freshly isolated primary BMC on a feeder layer. Conditioned medium was defined as the media recovered from feeder layer cultures alone after 24 hours. All PAH and TCDD exposures were tested after 24-hour incubation, unless otherwise noted.

### 2.5. Microarray

Microarray analyses were completed with triplicate WT and Cyp1b1-ko mice treated with either DMBA or TCDD as per above. Vehicle control animals were treated with olive oil. RNA was isolated using Qiagen's RNeasy mini-kit (Hilden, Germany) and quantified using a Nanodrop (Thermo Fisher Scientific; Waltham, MA) and integrity was assessed using formaldehyde agarose gel electrophoresis. Cy3 and Cy5 labeling was completed using Agilent Technologies' Dual Color Gene Expression kit and analysis was completed on the Whole Mouse Genome Microarray 4 × 44 slides, using the DNA Microarray Scanner and feature extraction software (Santa Clara, CA). Analysis was completed using EDGE3 software package [6, 22].

### 2.6. Treatment* In Vitro*

Freshly isolated primary BMC were plated at a density of 1.05 × 10^6^ cells/cm^2^ in fresh culture media (*α*MEM + 20% FBS). Coculture experiments were completed using feeder cells, while monocultures were completed with primary bone marrow cells on tissue culture-treated plastics. PAH, TCDD, or DMSO control treatments were added to the cell suspensions at plating. Cells were treated for 24 hours prior to analysis.

### 2.7. CFU

Colony Forming Unit (CFU) assays were completed as per manufacturer's instructions. In brief, BMC were isolated by centrifugation either directly from femur extraction/bone marrow isolation or after 24-hour treatment. The BMC were resuspended in fresh culture media at 5 × 10^5^ cells/mL for PreB progenitors. 1/10 volume of cells was mixed with the appropriate methocult media (Stem Cell Technologies, Vancouver, BC, Canada) and incubated at standard culture conditions (37°C at 5% CO_2_ in saturated atmospheric humidity) for 7 days. Colonies were counted by visual inspection via light microscopy. Statistical significance was determined by ANOVA with a Tukey* post hoc *test for multiple comparisons, *p* < 0.05 (GraphPad Prism, San Diego, CA).

### 2.8. qPCR

Induction of* Cyp1b1 *was determined using qPCR analysis. Total RNA was isolated as described above. Reverse transcription was completed using random oligos and GoTaq, and specific expression was determined using SYBR qPCR master mix, as per manufacturer's instructions (Promega, Corp; Madison, WI). Signal was detected and integrated using the BioRad CFX Real Time PCR Detection System (Hercules, CA). Primers were obtained from IDT (Coralville, IA):* Cyp1b1 *(F: CCACTATTACGGACATCTTCGG, R: CACAACCTGGTCCAACTCAG) and* actin* (F: CAACGAGCGGTTCCATG, R: GCCACAGGATTCCATACCCA). Analysis was completed using GraphPad Prism (La Jolla, CA) software. Ct values were normalized to* actin* expression, fold change was calculated for induction by DMBA treatment, and interval range was calculated for graphical presentation. Fold change data were log transformed and analyzed for statistical significance by ANOVA with Tukey* post hoc* tests.

### 2.9. Residual PAH Extraction and HPLC Analysis

OP9 cells were cultured for 24 hours with media supplemented with 3 *μ*M DMBA. The media were collected and treated with *β*-glucuronidase (Sigma) at 2000 U/mL to optimize recovery of the lipid-soluble residual PAHs. Samples were spiked with an extraction control standard (dibenzo[a,l]pyrene) (NCI Chemical Carcinogen Reference Standard Repositories, Midwest Research Institute, Kansas City, MO) prior to extraction using a 2 : 1 ethyl acetate : acetone (Sigma) organic phase, with repeated extraction centrifugation. The organic phase was dried under liquid nitrogen and the extracted compounds were stored at −80°C until analysis. Dried samples were resuspended in 100% methanol immediately prior to injection onto the HPLC column. Individual PAHs were separated using a Waters (Milford, MA) 2695 HPLC instrument with a C_18_ column, employing both a UV detector (wavelength 254 nm) and a Waters 470 scanning florescence detector. Data was collected with Waters Empower 3 software. Peaks were separated under a 50–100% methanol gradient over a period of 55 minutes [23].

## 3. Results

### 3.1. *In Vivo* Effects of DMBA and BP on PreB Lymphoid Progenitors* In Vivo*

**Figure 1:**
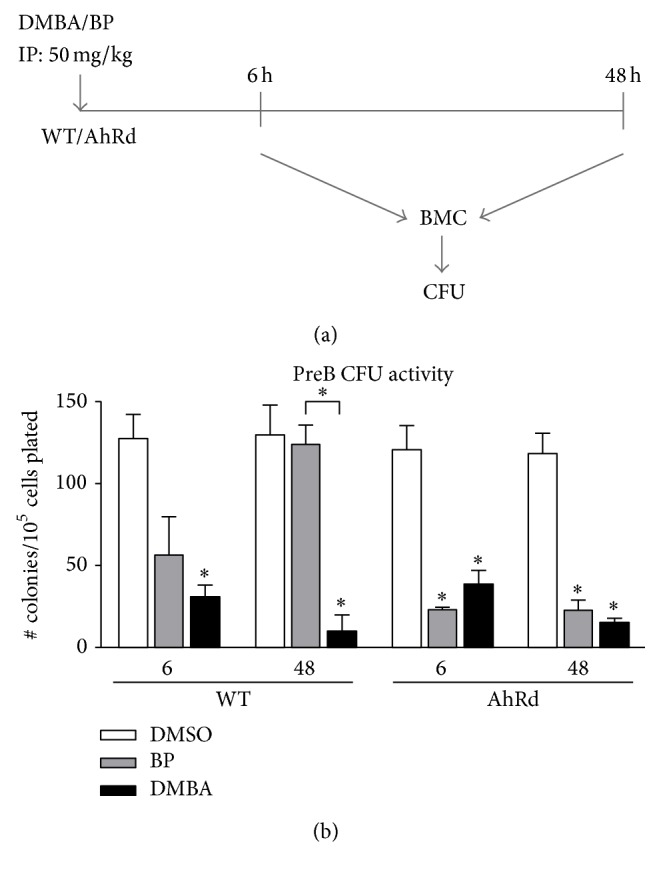
Comparison of progenitor activity after* in vivo* PAH treatment in mice with normal (WT) and diminished (AhR^d^) AhR activity. (a) Diagram of* in vivo* treatments and isolation points for colony forming unit (CFU) analyses. Mice were treated with DMBA or BP (50 mg/kg) or an equivalent volume of solvent control (DMSO) via a single IP injection. Bone marrow cells (BMC) were isolated 6 and 48 hours after treatment and analyzed for PreB CFU activity. (b) Comparison of PreB CFU activity in WT and AhR^d^ BMC 6 and 48 hours after PAH administration (50 mg/kg). Significance (^*∗*^ *p* < 0.05) indicates PAH suppression from untreated control or between PAH treatments at the same time point within a genotype, as indicated.

We have previously used an acute dosing animal model to study the effects of PAHs on bone marrow hematopoietic lineages [5, 6, 24]. Here, we have used PreB CFU assays to measure the suppression of lymphoid progenitors by IP administration of two well-characterized PAHs (BP and DMBA, 50 mg/kg). We examine the effects after 6 and 48 hours, which correspond to, respectively, the primary hepatic clearance and the midpoint of slow release from peritoneal fat (Figure 1(a)) [5, 6]. The primary clearance is enhanced by the induction of Cyp1a1 in the liver. The clearance kinetics are not significantly different between DMBA and BP [24, 25]. After 6 hours, DMBA and BP lower PreB CFU activity by 50–70 percent. After 48 hours, these activities change in opposite directions. DMBA treatment further declines to 90 percent suppression, while BP treatment shows a complete recovery (Figure 1(b)). This difference is observed in the mature cell populations after 48 hours, wherein there is 50 percent suppression of total BMC by DMBA, but there is no suppression by BP. The progenitor changes depend on Cyp1b1 expression and extend to B and T lymphocytes in the spleen and thymus, which derive from these BM progenitors [6, 26].

In AhRd mice, where DMBA and BP fail to activate the AhR, both PAHs reach the same peak suppression levels within 6 hours (Figure 1(b)). BP, in contrast to DMBA, exhibits an AhR-mediated recovery effect on BMC progenitor activity, likely due to the ready formation of BP quinones [5]. Elsewhere, we have shown that these effects of DMBA are dependent on Cyp1b1 [5, 6, 24]. Cyp1a1 deletion also prevents this recovery [25, 27].

### 3.2. Reconstitution of an* In Vitro* Model

**Figure 2:**
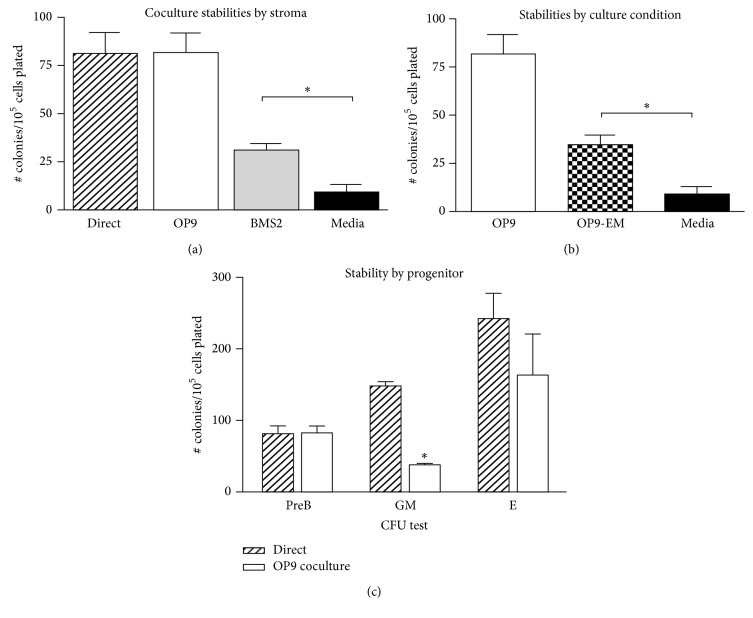
Conditions that sustain progenitor colony forming activities in collagenase-released bone marrow cells (BMC). (a) Comparison of PreB CFU activity in BMC maintained alone in culture media for 24 hours after isolation (media) or sustained by being cocultured for this period with an adherent monolayer of either OP9 cells or BMS2 cells. Direct cultures were placed in the methocult media immediately upon BMC isolation. ^*∗*^ *p* < 0.05. (b) BMC PreB CFU activity following 24-hour culture in media alone, in coculture with OP9 cells, or with media enriched by 24-hour preculture with OP9 cells (OP9-EM).  ^*∗*^Statistical significance; *p* value < 0.05. (c) CFU progenitor cell proliferative activity (PreB, granulocyte-monocyte (GM), and erythroid (E)) in BMC, when directly isolated or after 24 hours in OP9 coculture. ^*∗*^ *p* < 0.05 for comparison between direct BMC culture and OP9 coculture.

To better assess the mechanism of PAH effects on BM stem and progenitor cells, we have established an* in vitro* model. The first step was to sustain the BMC activity for a sufficient exposure period to see PAH effects (24 hours). BMC isolated by collagenase treatment provide extensive CFU activity when assayed directly in the methylcellulose support media of the progenitor clonal expansion assay. However, 24-hour culture in media alone, prior to transfer to the CFU assay media, resulted in a near complete loss of each CFU activity (representative example, Figure 2(a)). To sustain progenitor activities for 24 hours, we tested the capability of coculture with mesenchymal multipotential stromal lines. BMS2 cells, which were derived from continuous culture of adherent mouse BM progenitor cells [20], retained 40 percent of the activity. OP9 cells, derived from the AGM region of mouse embryos [18], have been extensively used to sustain stem cell activity [28, 29]. OP9 cells fully sustained progenitor activity for 24 hours (Figure 2(a)). C3H10T1/2 cells, which were derived from mouse embryo fibroblasts [21], also appreciably sustained PreB CFU progenitor cell activity (data not shown). Culture medium enriched by OP9 cells (OP9-EM) for 24 hours prior to culture was comparably effective as BMS2 cells in maintaining BMC progenitor activity in absence of the OP9 support (Figure 2(b)).

We also used the CFU assays designed for granulocyte-monocyte (GM) and erythrocyte (E) progenitor populations (Figure 2(c)). The erythrocyte progenitor CFU activity was sustained almost as well as PreB CFU activity. In contrast, the GM progenitor cells lost most of their activity. This may be explained by the fact that OP9 cells do not express macrophage colony stimulating factor (mCSF) [30]. Addition of this factor, however, causes a loss of the other hematopoietic lineages [31].

**Figure 3:**
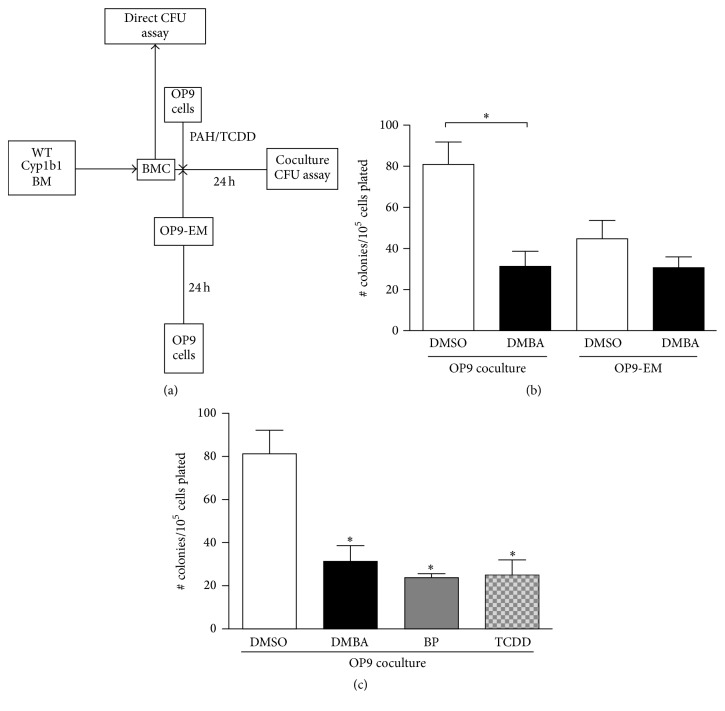
Effect of PAH treatment on WT progenitor activity in OP9 coculture from the fresh BMC preparation without* in vitro *culture. (a) Experimental design for assessing effects of PAHs on progenitor proliferation (PreB CFU activity) of cultured BMC. Cells isolated from the BM of mice of different genotypes are cocultured with serum media for 24 hours alone, on a monolayer stromal support (OP9 or BMS2 cells) or media enriched by 24-hour culture with OP9 cells (OP9-EM). PAH (1 *μ*M), TCDD (0.01 *μ*M), or an equivalent volume of DMSO solvent is added at the beginning of the 24-hour period. BMC (5 × 10^5^ cells) are added to the CFU methyl cellulose suspension assay at the end of 24 hours. Direct addition of BMC to the CFU assay provides a 100 percent reference activity. (b) Effect of DMBA (1 *μ*M) in OP9 coculture compared with effect in enriched medium (OP9-EM).   ^*∗*^Statistical significance; *p* value < 0.05. (c) OP9 coculture experiments with DMBA (1 *μ*M), BP (1 *μ*M), and TCDD (0.01 *μ*M) treatments showed significant (^*∗*^ *p* < 0.05) loss of activity after 24-hour treatment, as compared with the DMSO solvent control.

This OP9/BMC coculture model was then used to test the effects of PAHs on BMC progenitor activity (Figure 3(a)). BMC were either cultured with OP9 cells and serum-containing media or with OP9-EM media. The OP9/BMC coculture model was equally sensitive to both PAHs (BP and DMBA, each 1 *μ*M) and TCDD (10 nM) treatment, resulting in significant loss of PreB progenitor activity (Figures 3(b) and 3(c)). However, when OP9-EM was used with BMC instead of OP9 cells themselves, the response to DMBA was low (Figure 3(b)). The response to DMBA, therefore, derives, mostly from the OP9 cells, and the metabolism of DMBA by the AhR-inducible Cyp1b1 in hematopoietic BMC is ineffective. However, this experiment does not rule out effects mediated by AhR induction of inhibitory factors in OP9 cells.

**Figure 4:**
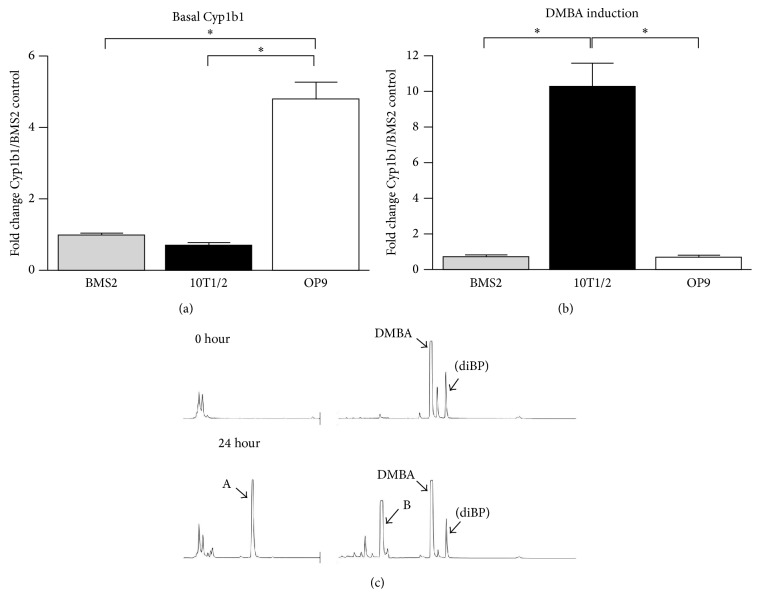
Cyp1b1 induction and DMBA metabolism in OP9 cells compared to other mouse multipotential progenitor lines. (a) Comparison of basal Cyp1b1 mRNA expression in OP9, C3H10T1/2 (10T1/2) cells, and BMS2 cells (set to 1.0). ^*∗*^ *p* < 0.05. (b) DMBA (24 hours, 1 *μ*M) induction of Cyp1b1 mRNA in 10T1/2 cells but not BMS2 and OP9 cells. ^*∗*^ *p* < 0.05. (c) HPLC separation of DMBA metabolites after zero- and 24-hour incubation of 3 *μ*M DMBA with OP9 cells (A: unresolved 8,9- and 10,11-dihydrodiols; B: unresolved 7- and 12-hydroxylmethyl). DiBP is added as an extraction standard after termination of the incubation.

A likely possibility is that DMBA is metabolized to active metabolites in OP9 cells. Since BMS2 cells express basal Cyp1b1 that is resistant to AhR induction, we tested OP9 cells for similar expression.  Figure 4(a) shows that Cyp1b1 mRNA is present in basal OP9 cells at higher levels than in BMS2 cells. C3H 10T1/2 cells had lower than normal basal levels, but were induced tenfold by DMBA. BMS2 and OP9 shared the same resistance to AhR induction (Figure 4(b)). This basal Cyp1b1 expression, with low AhR induction, is typical of the BM primary counterpart [3]. DMBA products seen here in the HPLC profile are unresolved 10,11- (major) and 8,9- (minor) dihydrodiols (peak A) and mixed phenols (peak B), which are typically seen for Cyp1b1 after a 3-hour incubation with DMBA [32] (Figure 4(c)). This activity in the OP9 cells strongly suggests that metabolism of DMBA in these cells contributes to their requirement for DMBA suppression of CFU activity.

### 3.3. The Role of Cyp1b1 in Maintaining PreB CFU in BMC

**Figure 5:**
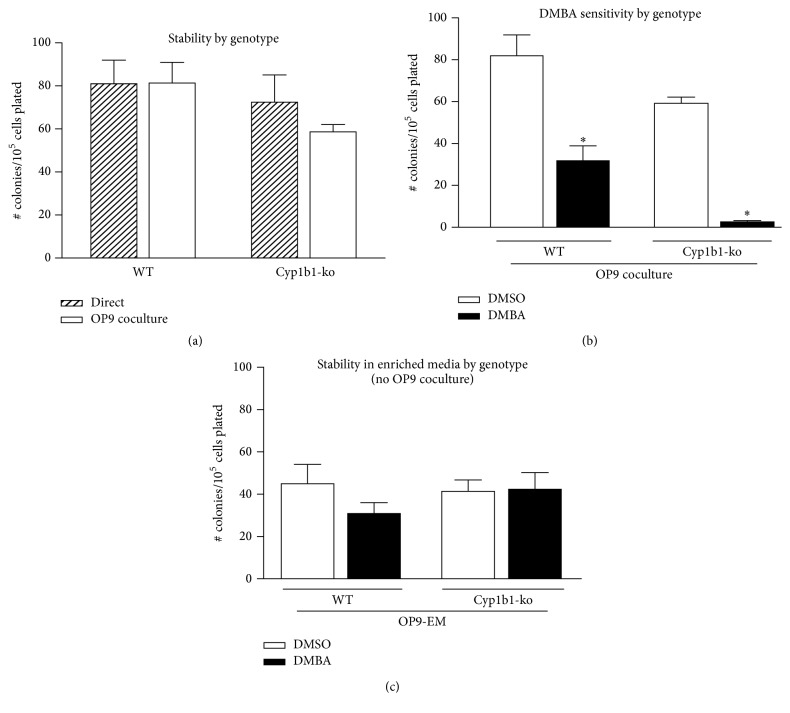
Comparison of WT and Cyp1b1-ko BMC with respect to PreB CFU progenitor cell activity. (a) Comparison of PreB CFU activity from BMC immediately after isolation (direct) and after 24-hour OP9 coculture. (b) Comparison of the PreB CFU activities for BMC after 24-hour solvent control (DMSO) and DMBA (1 *μ*M) treatments, using OP9 coculture conditions. ^*∗*^ *p* < 0.05. (c) Comparison of the PreB CFU progenitor cell activity for WT and Cyp1b1-ko BMC after 24-hour solvent control (DMSO) and DMBA (1 *μ*M) treatments in OP9 enriched medium (OP9-EM).

To test the contribution of Cyp1b1 to the PreB CFU activity, we cocultured Cyp1b1-ko BMC with OP9 cells for 24 hours (Figure 5(a)). The CFU activity of Cyp1b1-ko BMC, directly following the collagenase treatment, showed a 25 percent decline after the 24-hour coculture when compared to WT BMC (*p* = 0.07). Treatment of the coculture with 1 *μ*M DMBA indicated that the progenitor activity in Cyp1b1-ko was far more sensitive to DMBA than in WT BMC (62% versus >90% inhibition) (Figure 5(b)). This reinforces the concept that DMBA sensitivity of BMC in this coculture model primarily derives from OP9 cells, likely from conversion by Cyp1b1 reactive dihydrodiol epoxide metabolites. Accordingly, the OP9-enriched media (OP9-EM) were partially effective in supporting PreB CFU in WT and Cyp1b1-ko BMC (Figure 5(c)). In each case, there was no effect of DMBA in absence of the OP9 cells.

The much enhanced sensitivity of Cyp1b1-ko BMC suggests that Cyp1b1 provides stability by regulating constitutive progenitor/stem cell support processes of the hematopoietic cells themselves. We have previously shown that Cyp1b1 prevents oxidative stress, which is highly toxic to these hematopoietic cells [13].

### 3.4. Role of Cyp1b1 in OP9 Cells

**Figure 6:**
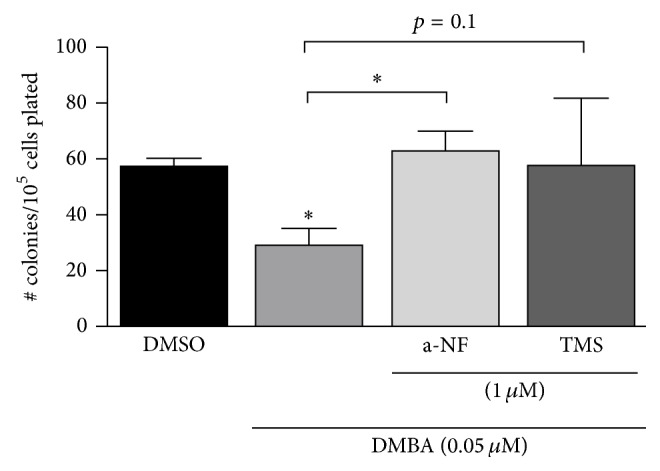
Reversal of DMBA suppression of PreB CFU activity from BMC by Cyp1b1 inhibitors, a-naphthoflavone (a-NF) and tetramethoxystilbene (TMS). To enhance sensitivity to DMBA, BMC from XPC−/− mice (low DNA repair) are used for the OP9 coculture. DMBA concentration is then decreased to 0.05 *μ*M, which is more sensitive to inhibitors. Inhibitors (1 *μ*M) were incubated with the cells for 24 hours prior to PreB CFU analyses. ^*∗*^ *p* < 0.05.

The activity of TCDD in this coculture system leaves open the possibility that DMBA may act directly on BMC progenitors via AhR activation in OP-9 cells. Tetramethoxystilbene (TMS) and *α*-NF each potently inhibit Cyp1b1. *α*-NF is also an antagonist for the AhR. We have used these inhibitors to test the participation of Cyp1b1 in DMBA-mediated PreB CFU suppression, which we have now shown can only arise from OP9 cells. One problem is that DMBA binds to Cyp1b1 very potently and is, therefore, difficult to competitively inhibit. In work to be described elsewhere, we have shown that the introduction of a deficiency in the DNA repair gene, XPC (XPC−/−) [33], into the BMC greatly enhances the potency of the PAH suppression (Rondelli, unpublished observation), which is now effective at 20 nM DMBA. This increase in potency allows complete inhibition by either a-NF or TMS (1 *μ*M each). OP9 cells, therefore, are functioning through Cyp1b1 (Figure 6).

### 3.5. Effects of Cyp1b1-ko on Constitutive and DMBA-Induced BM Gene Expression

Examination of gene array changes in BM,* in vivo*, after 12 and 24 hours indicated very few responses to DMBA, despite the stimulation of canonical AhR targets equal to that by either BP or TCDD and the extensive stimulation of chemokines and cytokines by BP [5, 6]. We interpreted the low response of DMBA to specific targeting of the small population of progenitor cells rather than the predominant mature cells. To understand the anomalous effects of Cyp1b1 and DMBA in the coculture model, we identified gene expression differences between WT and Cyp1b1-ko BMC (lower level of local metabolism). We also compared the* in vivo* effect of TCDD to determine whether deletion of Cyp1b1 can activate the AhR in the BM. This 12-hour treatment period corresponds to near maximal PreB CFU suppression.

Table 1 shows examples of gene expression levels in mRNA from BM cells of Cyp1b1-ko mice compared to WT mice. We show that genes, which fail to respond to DMBA in WT mice, now respond in Cyp1b1-ko mice, either up- or downregulated. All changes shown here are to some degree shared by TCDD. Four groups (A–D) represent stimulation; one group (E) represents suppression.

Group A genes, typified by Cyp1a1, Ahrr and Cyp1b1, are classical AhR responders, with DRE elements that show similar peak upregulation by each PAH at 12 hours [5]. DMBA strongly induces expression in Cyp1b1-ko mice.

Group B comprises examples of genes involved in inflammatory stress, which are stimulated in WT mice by TCDD, but which show larger increases with BP (within 6 hours) [5]. In WT mice, Group B genes show no stimulation with DMBA, even after 12 hours, thus distinguishing these genes from the classic Group A responders. Surprisingly, the expression of each gene in Cyp1b1-ko mice is appreciably enhanced by DMBA. This extra response compared to WT mice may arise because of increased DMBA concentration or a liver metabolite, when the BM lacks metabolism by Cyp1b1.

Like genes in Group B, genes in Groups C and D also respond to TCDD in WT mice, but not to DMBA. Each shows increased expression in Cyp1b1-ko mice. The inflammatory responses in Group B are enhanced by treatment of Cyp1b1-ko mice by DMBA and Group C responses are suppressed by DMBA treatment in Cyp1b1-ko mice, while Group D increases are unaffected by DMBA treatment. Group E genes exhibit decreased expression in WT mice treated with TCDD and in Cyp1b1-ko mice in the absence of treatment, which is also unaffected by DMBA. Essentially, Group E is the suppression counterpart of Group D. Each response to Cyp1b1-ko in Groups D and E is matched by TCDD in WT mice, but not by DMBA. Many of these responses are matched by similar genes in the same family, which are listed in Table 1 legend. The majority of genes in Groups C–E that are characterized by Cyp1b1-ko mice regulate developmental responses, including hematopoiesis.

## 4. Discussion

Recent* in vivo* studies of PAH effects on hematopoietic differentiation in the BM show that single doses rapidly suppress lymphoid, myeloid, and erythroid progenitor activities, when measured with the corresponding CFU assay. The shared suppression process and direct measurements of early progenitor and stem cell populations suggest that hematopoietic stem cell activity is targeted. The relationship of suppression effects to mature cell losses in BM, spleen, and thymus indicates that each of these cellularity losses arises, initially, from a PAH-induced failure to replenish mature cells from the BM hematopoietic stem cells [6]. Each of these tissues delivers mature B and T lymphocytes to the vascular and lymphatic circulation. The progenitor suppression is mediated by Cyp1b1, independent of AhR, despite appreciable induction of Cyp1b1 expression in the BM. We hypothesized that this anomaly arises because the key Cyp1b1 activity is provided by a BM mesenchymal progenitor subpopulation, wherein Cyp1b1 is not inducible.

In the present studies, we describe a coculture model that reproduces* in vivo* PAH suppression of BM progenitor cells* in vitro*. This model establishes the potential for involvement of BM mesenchymal Cyp1b1 in this suppression. Notably, we show that the PAH suppression can arise from Cyp1b1 in the supporting OP9 mesenchymal cells rather than from Cyp1b1 expressed in the BMC. OP9 cells share multipotential mesenchymal progenitor activity with the BM-derived, BMS2 cell line [29, 34–39]. Other mesenchymal lines that are derived from the AGM region of the embryo that yields the OP9 cells exhibit considerable selectivity with respect to this stem cell support capacity [35]. OP9 and BMS2 cells also share appreciable basal Cyp1b1 expression that, like the equivalent primary BM cells, scarcely responds to AhR induction (Figure 4). C3H10T1/2 cells, another multipotential mesenchymal line derived from mouse embryo fibroblasts, also supported the PreB CFU progenitor activity. However, they show high AhR-mediated induction of Cyp1b1.

The origin of the Cyp1b1 activity is most clearly demonstrated by the finding that OP9-enriched media alone sustain PreB progenitor activity in BMC but are largely resistant to DMBA, despite the expression of Cyp1b1 in the BMC. TCDD also suppresses PreB CFU in the BMC/OP9 coculture model (Figure 3(c)), thus indicating a contribution from direct activation of the AhR, which may extend to DMBA. TCDD is not metabolized and may, therefore, transfer into the CFU assay. The effective reversal of DMBA activity by Cyp1b1 inhibitors, a-NF and TMS (Figure 6), indicates that metabolism by Cyp1b1 is much more important than AhR activation.

Surprisingly, coculture of Cyp1b1-ko BMC with OP9 cells greatly increases the sensitivity to PAHs. This enhanced sensitivity probably arises because Cyp1b1 also protects against oxygen-induced oxidative stress (OxS) in endothelia and pericytes, which are each present in BM vascular cells [13]. Increased OxS in Cyp1b1-ko BMC may reach the progenitors and increase their susceptibility to the PAH metabolites. Preliminary studies show that decreasing DNA repair in the BMC population, through loss of XPC activity, similarly increases lymphoid progenitor susceptibility to these metabolites (Figure 6) [33].

This DMBA suppression of the progenitor cells most likely derives from metabolites transferred from the OP9 cells. It remains to be determined why Cyp1b1 in BMC is ineffective in DMBA activation compared to the Cyp1b1 in OP9 cells. The first approach to resolving this problem will be to compare the metabolism of DMBA in these fractions. DMBA metabolites generated by Cyp1b1 in the OP9 cells may also remove progenitor support factors secreted by these cells. DMBA addition, during the enrichment pretreatment, was relatively ineffective in diminishing the activity of the enriched media (EM factors) (not shown). Such factors appear to replace about half of the progenitor support activity in OP9 cells (Figure 3(b)). The remaining support comes from labile factors, which are not retained in the OP9-EM.

*In vivo*, BP-mediated suppression of progenitor activity is restored after approximately 6 hours. We have shown that this reversal derives from metabolic activation generated by AhR-mediated induction of Cyp1a1 [5]. Most likely, BP quinones produced in the liver upregulate cytokines and chemokines in BM through NF-kB activation. Cytokines, like IL6, or the antiapoptotic NF-kB effects can selectively reverse the BP toxicity on progenitor cells. This reversal of CFU suppression is not seen with BP treatment in the OP9 coculture model, where BP has comparable effects to DMBA. This parallel is seen* in vivo*, in AhRd mice, which are resistant to PAH induction (Figure 1).

Gene expression analyses show that Cyp1b1 deletion in the BMC does much more than remove PAH metabolism.* In vivo*, effects arising from endogenous or dietary substrates are evident following the BMC deletion of Cyp1b1. Additionally, deletion of Cyp1b1 introduces* in vivo* effects of DMBA that are absent in WT mice. Such effects arise either from increased DMBA in the BM or new alternative metabolites, which can each result from removal of the Cyp1b1 metabolism. The set of chemokines and cytokines that were increased by BP and, to a lesser extent, by TCDD are elevated constitutively in Cyp1b1-ko BM. This activity is augmented by cotreatment with DMBA, which has no such effect in WT mice. The Cyp1b1 deletion alone did not increase the canonical AhR/ARNT-directed transcription. The AhR-type activity in Cyp1b1-ko BM, therefore, does not derive from AhR/ARNT complexes. We have previously concluded that the acute cytokine/chemokine responses to BP, which are only partially reproduced by TCDD, actually arise from a partnership between AhR and NF-kB transcription factors. A stimulatory partnership between AhR and Rel B increases cytokine expression [40].

The gene expression changes caused by Cyp1b1-deletion in Table 1 arise from a loss of the effects of endogenous and dietary compounds that affect BM gene expression and from changes in the ratios of the BM hematopoietic cell populations. Stem cells and hematopoietic lineage progenitors are present in low proportions and, therefore, will contribute directly to gene expression. Physiological intervention by Cyp1b1 deletion occurs through removal of estradiol and generation of hydroxyl products, which variously target nuclear (ER*α*, ER*β*) and membrane associated receptors (ER*α*, GPER), and affects an increasing number of processes, including blood pressure and cancer [41] and tryptophan dimerization products [42]. We detected many effects of Cyp1b1 deletion on developmental factors. Even though expressed at low levels, these genes can only be detected if appreciably expressed in more abundant cell types among the many present isolated from BM (perhaps >5 percent of total). By far the predominant response pattern observed is an increase or decrease in Cyp1b1-ko mice with a correspondingly diminished response in Cyp1b1-ko mice treated with DMBA. Creb3l3, Hoxb6, Hoxb9, Sox15, and Sox19 are each appreciably increased in Cyp1b1-ko mice through a mechanism that is shared by TCDD and BP in WT mice but is blocked by DMBA in Cyp1b1-ko mice. Some Cyp1b1-ko responses are unaffected by DMBA, including stimulations of Socs3 and Nr4a1, which are similarly increased by TCDD, and decreases in Rag1 and Spp1/osteopontin. These gene changes point to a novel crosstalk between the effects of endogenous Cyp1b1-ko signaling and the AhR that is selectively affected by DMBA.

## Additional Points

*Highlights*. The selective suppression of hematopoietic progenitor proliferation (CFU activity) by DMBA is directed by metabolism in BM stromal cells, which exhibit robust basal expression of Cyp1b1 but minimal induction. OP9 mouse embryo mesenchymal cells secrete factors that support proliferation of BM lymphoid and erythroid progenitors for at least 24 hours. This support is substantially replicated by OP9-enriched media and by BMS2 and C3H10T1/2 cells. Cyp1b1 is expressed at sufficient levels in OP9 cells to generate reactive DMBA metabolites that inhibit progenitor cell expansion. Cyp1b1 levels are much lower in isolated BMC and insufficient to generate inhibitory metabolites. Cyp1b1 provides basal protection to progenitors in BM, either through removal of oxidative stress or through the observed suppression of the AhR/NF-kB stimulation of chemokines and cytokines. These secreted proteins can protect progenitor cells or the activity of precursor hematopoietic stem cells.

## Figures and Tables

**Table 1:** Genes targeted by Cyp1b1 endogenous substrates and AhR. Separation by DMBA responses.

Gene	WT (FC)		Cyp1b1-ko (FC)		Set
	TCDD	DMBA	Control	DMBA	
*Stimulated*					

Cyp1b1	3.5	2.5	n/a	n/a	A
Cyp1a1	9.7	15.0	nd	[11.0]^g^	
Ahrr	3.8	4.1	nd	[10.0]^g^	

IL6	3.0	ns	2.5	4.5	B
Tnfa	2.0	ns	1.5	3.0	
Cxcl10^a4^	2.0	ns	1.5	4.0	
Egr2^a1^	2.9	ns	2.5	4.4	

Hbb-b1^a2^	2.4	1.3	2.2	ns	C
Fau	2.0	1.5	2.2	1.3	
Alas2^b1^	1.7	ns	1.6	ns	
Creb3l3	2.9	ns	7.6	1.9	
Hoxb6^a5^	2.6	1.3	2.7	ns	
Sox19^a6^	1.5	ns	5.0	2.2	
Polr2a	5.0	ns	6.2	2.3	

Klf4	3.6	1.3	2.1	1.7	D
Nr4a1	3.6	ns	2.0	2.2	
Hspa-la^a3^	8.5	ns	2.6	3.0	
Clqc^a7^	1.8	1.4	2.8	2.4	
Socs3	2.0	1.5	2.0	2.8	

Rag1	−1.8	ns	−3.0^*∗*^	−3.0	E
Erdr1	2.5	ns	−2.4	−2.4	
Col5a3	−1.5	ns	−3.0^*∗*^	−2.0	
Faim3	−1.5	1.3	−2.9	−1.5	
Spp1	−3.0	1.6	−2.2	−1.9	

Fold-changes (FC) are all significant; *p* < 0.05.

ns: not significant.

n/a: not available.

nd: not detectable.

[FC]^g^: relative to Cyp1b1-ko control genotype effect on PAH.

^a^Comparable responses from family members (a1, Egr1; a2, Hbb-a1/a2; a3, Hspa-lb; a4, Cxcl9; a5, Hoxb9; a6, Sox15; a7, Gimap1).

^b^No response from family member, Alas1.

^*∗*^No significant difference from BP.
